# Healthcare Contacts Prior to Suicide by Those in Contact With Mental Health Services

**DOI:** 10.1192/bjo.2025.10085

**Published:** 2025-06-20

**Authors:** Marcos Del Pozo Banos, Keith Lloyd, Louis Appleby, Nav Kapur, Ann John

**Affiliations:** ^1^Swansea University, Swansea, United Kingdom; ^2^National Confidential Inquiry into Suicide and Safety in Mental Health (NCISH), Centre for Mental Health and Safety, School of Health Sciences, University of Manchester, Manchester, United Kingdom

## Abstract

**Aims:** People under mental health (MH) services’ care are at increased risk of suicide. We aimed to identify opportunities for suicide prevention and underpinning data enhancement in people with recent contact with MH services.

**Methods:** A population-based study of all who died by suicide in the year following an MH services contact in Wales, 2001–2015 (cases), paired with similar patients, with the same mental health diagnoses, who did not die by suicide (controls). We linked the National Confidential Inquiry into Suicide and Safety in Mental Health and the Suicide Information Database – Cymru with primary and secondary healthcare records. We present odds ratios and 95% confidence intervals (OR [95% CI]) of conditional logistic regression.

**Results:** We matched 1,031 cases with 5,155 controls. In the year before their death, 98.3% of cases were in contact with healthcare services, and 28.5% presented with self-harm.

A high proportion (98.3%) of cases were in contact with primary and secondary healthcare services in the year before their death. Compared with controls, cases were more likely to attend emergency departments (OR 2.4 [2.1–2.7]) and have emergency hospital admissions (OR 1.5 [1.4–1.7]); but less likely to have primary care contacts (OR 0.7 [0.6–0.9]), out-patient attendances (OR 0.2 [0.2–0.3]) and missed/cancelled out-patient appointments (OR 0.9 [0.8–1.0]).

A high proportion of cases presented to primary and secondary healthcare services with accidents, injury and poisoning, and especially self-harm – more so than controls (for self-harm, 28.5% of cases compared with 8.5% of controls; OR 3.6 [2.8–4.5]). This was particularly true for female patients admitted to hospital with injury and poisoning (OR 3.3 [2.5–4.5] in females compared with 2.6 [2.1–3.1] in males).

**Conclusion:** We may be missing existing opportunities to intervene across all settings, particularly when people present to emergency departments and hospitals, especially with self-harm. Intent underlying injury and poisoning events may be undisclosed, or recorded as undetermined or without specifying intent when they may in fact be self-harm, particularly in females. Efforts should be made to appropriately identify those who are self-harming, including by direct and non-judgmental questioning on presentation underpinned by staff training and awareness. Prevention efforts should focus on strengthening non-urgent and routine contacts (primary care and outpatients), responding to emergency contacts, and better self-harm care. This study also highlights the benefits of enhancing clinical audit systems with routinely collected data for data completeness, breadth, and depth.

